# Evaluation of signal transduction pathways after transient cutaneous adenoviral gene delivery

**DOI:** 10.1186/1471-2172-12-8

**Published:** 2011-01-21

**Authors:** Lars Steinstraesser, Michael Sorkin, Frank Jacobsen, Sammy Al-Benna, Marco Rainer Kesting, Andreas David Niederbichler, Jan-Michel Otte, Tobias Hirsch, Jadwiga Stupka, Hans-Ulrich Steinau, Matthias Schulte

**Affiliations:** 1 Laboratory for Molecular Oncology and Wound Healing, Department of Plastic Surgery, Operative Reference Centre for Soft Tissue Sarcomas, BG University Hospital Bergmannsheil, Ruhr University Bochum, Bochum, Germany; 2 Dept. of Oral and Maxillofacial Surgery, Klinikum Rechts der Isar der Technischen Universität München, München, Germany; 3 Dept. of Plastic, Hand and Reconstructive Surgery, Burn Center, Medizinische Hochschule Hannover, Hannorver, Germany; 4 Dept. of Gastroenterology, St. Josef - Hospital, Ruhr University Bochum, Bochum, Germany

## Abstract

**Background:**

Adenoviral vectors have provided effective methods for *in vivo *gene delivery in therapeutic applications. However, these vectors can induce immune responses that may severely affect the ability of vector re-application. There is limited information about the mechanisms and signal transduction pathways involved in adenoviral recognition. For optimization of cutaneous gene therapy it is necessary to investigate molecular mechanisms of virus recognition in epidermal cells. The aim of this study was to investigate the signal transduction of the innate immunity after adenoviral DNA internalization in keratinocytes.

**Methods:**

*In vitro*, keratinocytes were transfected with DNA, in the presence and absence of inhibitors for signalling molecules. *In vivo*, immunocompetent and athymic mice (n = 3 per group) were twice transduced with an Ad-vector.

**Results:**

The results show an acute induction of type-I-interferon after *in vitro *transfection. Inhibition of PI3K, p38 MAPK, JNK and NFkappaB resulted in a decreased expression of type-I-interferon. In contrast to immunocompetent mice, athymic mice demonstrated a constant transgene expression and reduced inflammatory response *in vivo*.

**Conclusion:**

The results suggest an induction of the innate immunity triggered by cytoplasm localised DNA which is mediated by PI3K-, p38 MAPK-, JNK-, NFkappaB-, JAK/STAT- and ERK1/2-dependent pathways. A stable transgene expression and a reduced inflammatory response in immunodeficient mice have been observed. These results provide potential for an effective adenoviral gene delivery into immunosupressed skin.

## Background

The skin is the largest organ of the body, accounting for about fifteen percent of our body weight, and covering the entire external surface. While many believe its role is merely as an external covering, the functions of the skin are far more complex. The skin consists of three main layers - the epidermis, the dermis, and the hypodermis, each with their own function. It is the epidermis, which creates a barrier to and protects from pathogens of the outside world. This highly specialised layer is mainly composed of keratinocytes, melanocytes and dendritic cells. Its accessibility and specific anatomical and biological properties make the skin a very interesting organ for *in vivo *and *ex vivo *gene therapy approaches. In case of cutaneous gene therapy, gene delivery can be easily controlled and the skin surgically excised if any side effects occur[1-3].

Keratinocytes, the predominant epidermal cell type, are responsible for establishing a physical barrier and guaranteeing the structural integrity of the epidermis[3]. As the epidermis is known to produce a variety of cytokines and growth factors, keratinocytes may also be engineered as bioreactors to secrete gene products which have local or systemic effects[4,5].

In most gene therapy applications, a "normal" gene is inserted into the genome of an individuals cell or tissue to replace an "abnormal" disease-causing gene. In addition, foreign, therapeutical active genes can be introduced in order to add any not normally in the body produced metabolite. On this basis, gene therapy can be a promising tool for the treatment of a wide variety of inherited as well as acquired disease including genetically inherited skin disorders, tumours, metabolic disorders and infectious diseases (e.g. *epidermolysis bullosa*, *xeroderma pigmentosum*, ichtyosis, porphyria, squamous cell carcinomas)[6-9].

Different methods for gene delivery can be pursued, depending on the desired application. The approach used to deliver DNA into the skin will have an influence not only on the efficiency of DNA delivery, but also on the level and duration of transgene expression[10,11].

A carrier molecule called vector must be used to deliver the therapeutic gene to the target cells. Based on viral and non-viral vectors, different applications for gene delivery have been developed in the last decades[12,13]. For transient transduction of target cells, adenoviral vector systems possess the highest effectivity and have been used in 23.9% of the official agency sources (Gene Therapy Advisory Committee (GTAC), Recombinant DNA Advisory Committee (RAC), etc.) registered clinical trials of gene therapeutical applications for different indications, such as cancer, infectious or monogenic diseases worldwide in the last two decades[14].

*Adenoviridae *are non-enveloped, double stranded (ds), linear desoxyribonucleic acid (DNA) viruses with a genome of 35-40 kb and a particle size of 70-100 nm[15,16]. The adenoviral genome is well characterised and comparatively easy to manipulate. Most adenoviruses cause mild diseases in immunocompetent human adults and by deletion of crucial regions of the viral genome the vectors can be rendered replication-defective, which increases their predictability and reduces unwanted side effects. Moreover, deleted regions of the viral genome can easily be replaced by foreign genomic material encoding the therapeutical active metabolite[17].

The process of adenoviral entry into the host is extremely efficient and has been intensively studied. Adenoviruses exhibit a wide host range *in vitro *and *in vivo*; this range was also seen in nondividing cells[18]. In addition, the well-defined and easily manipulated viral genome favours the development of adenoviral vectors for gene therapy applications[19]. This, together with information from the complete library of human DNA opened up extensive opportunities for gene therapy in medical and surgical specialities[20].

The major disadvantage of adenoviral vectors is that they can effectively induce the adaptive and innate immune response immediately after infection, leading to an induction of proinflammatory cytokines and chemokines in mice, primates and humans[21-23]. Activation of innate immunity is associated with a reduction in efficacy of gene delivery[11,23]. It may further cause significant inflammatory reactions leading to morbidity and mortality of the transduced host[21,22].

Newer generations of helper-dependent, gutted adenoviral vectors or adeno-associated viruses, which are depleted of almost all viral coding sequences[24], cause diminished adaptive immune responses to these vectors and improve the duration of gene transfer[25]. However, acute toxicity and diminished vector persistence provoked by the innate immune response remain the most significant barriers to clinical application of this promising technology[26]. To improve safety, efficacy and duration of adenoviral gene transfer it is necessary to explore the mechanisms by which adenoviruses trigger the innate immune response.

The detection of microbial components by pattern recognition receptors (PRRs) is one of the earliest defense mechanisms that is known to trigger innate immune responses against infections[27,28]. Of the many classes of molecules detected by cells as pathogen associated molecular patterns (PAMPs), nucleic acids are potent and broadly recognised[29-35]. To sense nucleic acids the immune system employs several classes of receptors, including RNA helicases that can respond to cytosolic ribonucleic acid (RNA) and DNA under certain conditions (retinoic acid-inducible gene I (RIG-I)/melanoma differentiation associated gene 5 (mda5)[35-37]), and Toll-like receptors (TLRs) that can recognise endosomal dsRNA (TLR-3)[38], singlestranded (ss)RNA (TLR-7/-8)[39-41], and hypomethylated DNA (TLR-9)[42].

Activation of TLRs occurs on the cell membrane surface (TLR-1, -2, -4, -5, -6 and -11) or within endosomal compartments (TLR-3, -7 and -9) and requires either a Myeloid differentiation primary response gene 88 (MyD88) or TIR-domain-containing adapter-inducing interferon-β (TRIF) adapter molecules[43]. These proteins facilitate activation of downstream signalling cascades, which lead to the activation of inflammatory transcription factors, including nuclear factor-kappa B (NFκB), activator protein 1 (AP-1) and interferon regulatory factors (IRF)[43,44]. The presence of viruses in cells is detected by TLR-3, -7, -8 and -9. TLR-3 recognises double-stranded (ds) ribonucleic acid (RNA)[45], TLR-7 and TLR-8 bind single-stranded RNA[46,47], whereas TLR-9 senses dsDNA which is also contained in adenoviruses[42].

Since the discovery of TLR-9, there has been a growing body of evidence that DNA derived from microbial and host cells can be recognised via a TLR-9-independent mechanism. DNA recognition in these pathways is sequence independent and occurs in the cytoplasm of the cells[48,49]. Different ligands, such as adenoviral, mammalian and vertebrate DNA as well as dsDNA have been characterised for TLR-independent recognition in antigen presenting cells (APCs)[50-57].

Recently, a DNA sensor and activator of innate immune responses has been identified and termed DNA-dependent activator of IFN-regulatory factors (DAI and also known as DLM-1 and ZBP1)[58]. Subsequent studies have shown the presence of an additional mechanism(s) for DNA-sensing and activation of the innate immune system, as well as a mechanism of negative regulation of cytosolic DNA-mediated immune responses[59,60]. NACHT-leucine-rich repeat-PYD containing protein 3 (NALP3) also known as cryopyrin, and its adaptor protein apoptosis-associated speck-like protein containing a CARD (ASC), regulate secretion of interleukin (IL)-1β in response to an adenovirus infection[61,62]. Inflammasome activation also occurs upon cytosolic exposure of DNA, though in this case DNA-sensing was shown to be dependent on ASC and not NALP3[61]. It remains unclear, however, as to whether the NALP3/ASC inflammasome can directly detect cytosolic DNA or if the response is triggered by another protein. Another negative regulator of the DNA-mediated innate immune response, adenosine deaminase acting on RNA 1 (ADAR1), has also been reported[60]. ADAR1 is an IFN inducible protein that possesses DNA-binding domains similar to those found in DAI.

Several studies on (adenoviral) DNA induced innate immune reaction have focused on antigen presenting cells (APCs) such as dendritic cells (DCs) or macrophages (MΦ). However, information about innate immune reactions and mechanisms in virus recognition in epithelial cells, such as keratinocytes, is very limited. For optimization of cutaneous gene therapy, it is necessary to investigate the immune reaction of the predominant epidermal cell type after adenoviral challenge. Since keratinocytes represent the majority (approx. 90%) of cells of the outermost epidermal layer[63], the aim of this study was to investigate the signal transduction of the innate immunity after adenoviral DNA internalization in this specific cell type. In addition, systemic effects of adenovirally induced immune reactions will be studied.

## Results

### DNA internalization induced innate immune reaction

In order to get a deeper insight into the molecular mechanisms of the innate immune system after (adenoviral) DNA internalization into keratinocytes, we decided to use a GFP-encoding adenovirus for this study. Since this generation of adenoviral vectors exhibit a high immunogenicity, this vector seemed to be an interesting tool for the investigation of signal transduction moleculess involved in adenovirus detection.

**Figure 1:**
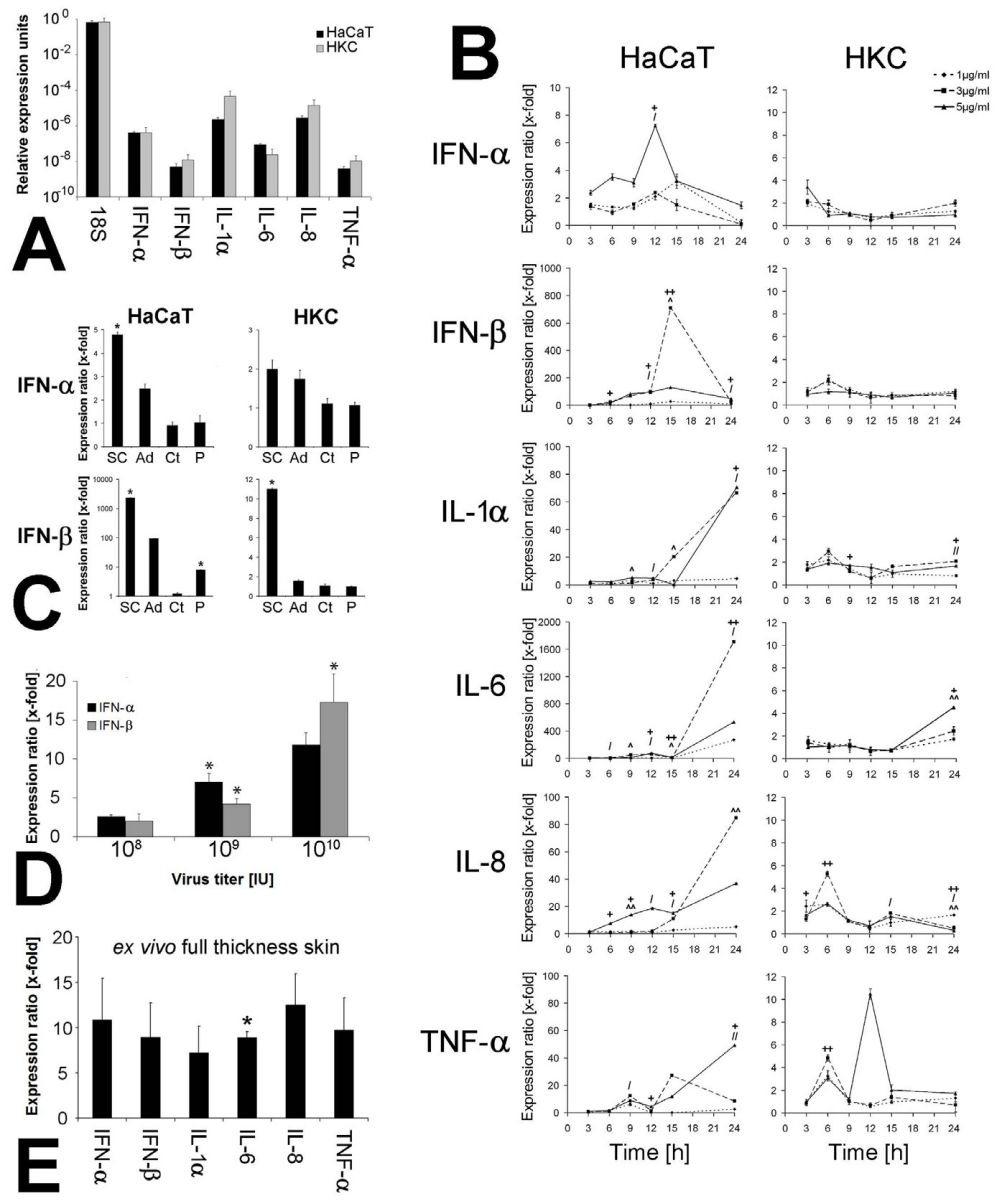
**DNA internalization induced innate immune reaction**. (A) RT-PCR analysis of type-I-interferon and cytokine basal expression in untreated HaCaT cells and HKC. (B) RT-PCR analysis of type-I-interferon and cytokine expression in HaCaT cells and HKC stimulated with different doses of adenoviral DNA (1, 3 and 5 μg DNA/ml medium) for 3 to 24 h. Results were normalized to a vehicle control (Fugene alone) (^ = p < 0.05, ^^ = p < 0.005 (1 μg DNA/ml medium);/= p < 0.05,//= p < 0.005 (3 μg DNA/ml medium); + = p < 0.05, ++ = p < 0.005 (5 μg DNA/ml medium). Data was presented as mean ± SEM (n = 3 per group). (C) RT-PCR analysis of type-I-interferon expression in HaCaT cells and HKC stimulated with DNA (5 μg DNA/ml medium) from adenovirus (Ad), *Saccharomyces cerevisiae *(SC), calf thymus (CT) and plasmids (P). Cell were stimulated for 6 h (HKC) and 15 h (HaCaT cells). Results were normalized to a vehicle control (Fugene alone). Data was presented as mean ± SEM (n = 3 per group), * = p < 0.05). (D) RT-PCR analysis of type-I-interferon of HaCaT cells transduced with different concentrations of adenovirus (10^8 ^- 10^10 ^IU; * = p < 0.05). (E) RT-PCR analysis of type-I-interferon and cytokine induction in human full thickness skin 12 h after injection of 10^10 ^IU of an adenoviral type 5 vector encoding for the green fluorescent protein (GFP) (* = p < 0.05). Results were normalised to 18S ribosomal RNA (ratio: target gene/housekeeping gene) and compared to non-treated samples (vehicle control).

The basal mRNA levels of 18S ribosomal RNA, type-I-interferons and cytokines (IL-1α, IL-6, IL-8 and TNF-α) possessed a comparable profile in HaCaT cells HKC (Figure 1A). HKC possessed significant higher levels of IL-1α (20-fold, p = 0.014) and IL-8 (5-fold, p = 0.002) mRNA compared to HaCaT cells. In contrast, mRNA level of IL-6 was detected on a significant higher level in HaCaT cells (3.7-fold, p = 0.045).

In order to get any information whether cytoplasmatic localised adenoviral DNA is capable to induce an innate immune reaction, human keratinocytes were transfected with DNA. Adenoviral DNA internalization led to a significantly increased (p = 0.007) IFN-α expression 12 h post transfection in HaCaT cells. The same cells demonstrated a significant increase in IFN-β expression (700-fold at stimulation with 3 μg DNA/ml medium, p = 0.002) after stimulation for 15 h (Figure 1B).

Besides type-I-IFN expression, there was significant increase in expression of inflammatory cytokines, namely interleukin (IL)-1, IL-6, IL-8 and TNF-α recorded in HaCaT cells and HKC with a maximal induction 24 h (HaCaT) and 12 - 24 h (HKC) post transfection (Figure 1B).

After detection of an adenoviral DNA induced inflammatory response in human keratinocytes it was interesting to see whether DNA from other species is also capable to activate an inflammatory reaction in these cells. RT-PCR analysis showed an increased induction of IFN-α in both cell types after *S. cerevisiae *(SC-) and Ad-DNA treatment (Figure 1C). Maximal induction of IFN-α after treatment with SC-DNA was 4.8-fold in HaCaT cells and 2-fold in HKC. Transfection of calf thymus (CT-) and plasmid (P-) DNA did not show any significant effect in either cell type. The transfection with SC-DNA led to a significant increase (p < 0.05) of IFN-β in both cell types. In HaCaT cells, a 2380-fold upregulation was detected (p = 0.001) whereas HKC accounted for a 11-fold increase of transcription (p = 0.007). Ad-DNA stimulation of both cell types also induced an expression of IFN-β in keratinocytes (94-fold in HaCaT cells; 1.6-fold in HKC). No changes in IFN-β expression of both cell types were demonstrated post CT-DNA stimulation and P-DNA induced upregulation of IFN-β was only detected in the HaCaT cell line (8-fold change; p = 0.012).

In order to get any information about the innate immune reaction after adenoviral transduction, HaCaT cells were transduced with different concentrations of adenoviral vectors (10^8 ^- 10^10 ^infection units (IU) of the GFP-encoding adenoviral vector (Figure 1D). A concentration dependent induction of IFN-α and IFN-β was observed 15 h post transduction (IFN-α: 2.6-fold (10^8 ^IU, p = 0.056), 7-fold (10^9 ^IU, p = 0.02), 11.8-fold (10^10 ^IU, p = 0.164); IFN-β: 2-fold (10^8 ^IU, p = 0.171), 4.2-fold (10^9 ^IU, p = 0.046), 17.3-fold (10^10 ^IU, p = 0.0156).

An adenoviral transduction of human *ex vivo *full skin samples led to an induction of IFN-α (11-fold; p = 0.162); IFN-β (10-fold; p = 0.168); IL-1α (7-fold; p = 0.167); IL-6 (9-fold; p = 0.001); IL-8 (13-fold; p = 0.076) and TNF-α (10-fold; p = 0.133) (Figure 1E).

### Involvement of Toll-like receptor family in DNA recognition

**Figure 2:**
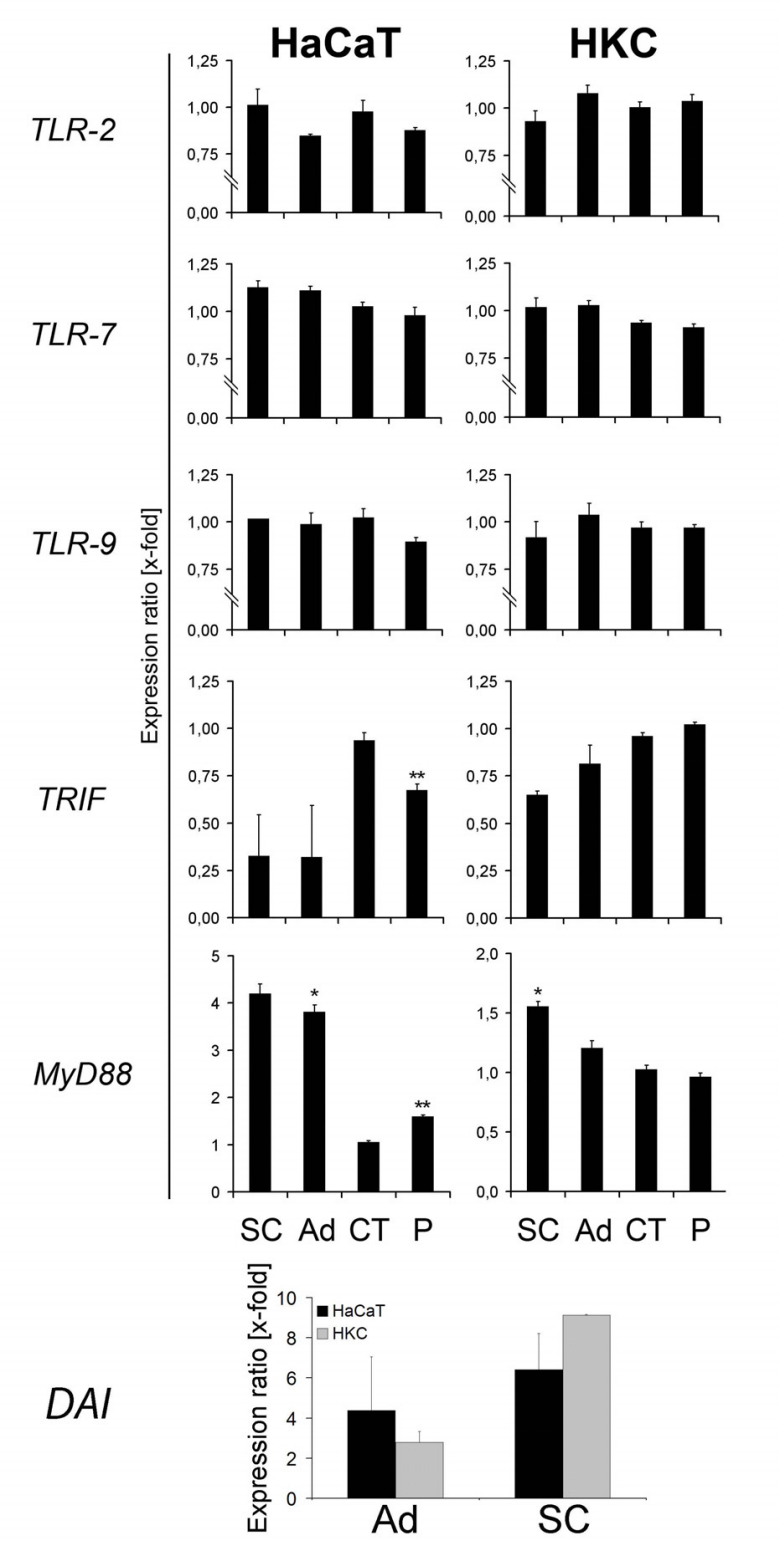
**Involvement of Toll-like receptor family in DNA recognition**. RT-PCR analysis (45 cycles) for TLR-2, -7, -9, TRIF and MyD88 and DAI expression in HaCaT cells and HKC stimulated with DNA (5 μg DNA/ml medium) from adenovirus (Ad), *Saccharomyces cerevisiae *(SC), calf thymus (CT) and plasmids (P). Cells were stimulated for 6 h (HKC) and 15 h (HaCaT cells). Data from DAI expression was generated from cells transfected with 5 μg DNA/ml medium. Results were normalised to 18S ribosomal RNA (ratio: target gene/housekeeping gene) and compared to non-treated samples (vehicle control) (* = p < 0.05; ** = p < 0.005 to vehicle control).

Toll-like receptors (TLR) are among the most important receptor of innate immunity. For an investigation of the involvement of TLRs in adenoviral DNA detection. The expression levels of TLR-2, -7 and -9 were determined after transfection of DNA from different species. There was no significant change in expression of TLR-2, -7, -9 and TRIF in both cell types (Figure 2). An increased amount of MyD88 mRNA was detected in HaCaT cells stimulated with SC-DNA (4.2-fold; p = 0.057), Ad-DNA (3.8-fold; p = 0.041) and P-DNA (1.6-fold; p = 0.001). An increased amount of MyD88 mRNA was detected in HKC cells stimulated with SC-DNA (1.6-fold; p = 0.03) and Ad-DNA (1.2-fold; p = 0.3). Stimulation of HaCaT cells and HKC with CT-DNA and P-DNA stimulation of HKC did not show any significant differences.

A transfection of HaCaT cells with Ad- and SC-DNA led to an induction of DAI. Transfection with Ad-DNA led to a 4.3-fold (HaCaT, p = 0.15) and 2.79-fold (HKC, p = 0.059) increase of DAI mRNA. Moreover, treatment of the cells with SC-DNA resulted in a stronger induction of DAI (6.4-fold (HaCaT, p = 0.087), 9.1-fold (HKC, p = 0.13)).

### DNA recognition and signal transduction

**Figure 3:**
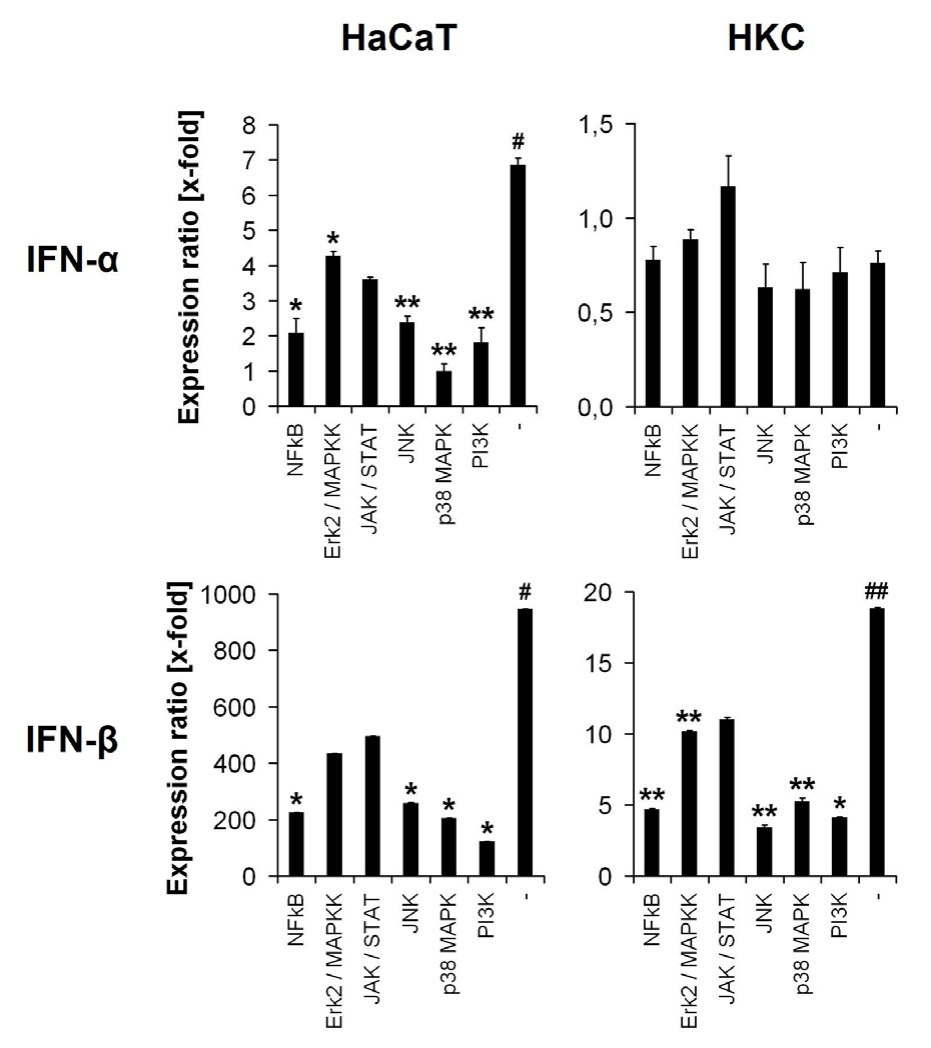
**DNA recognition and signal transduction**. RT-PCR analysis of type-I-interferon expression in HaCaT cells and HKC after preincubation with 10 μM of NFκB-, Erk2/MAPKK-, JAK/STAT-, JNK-, p38 MAPK- and PI3K-inhibitors (Sigma, Steinheim, Germany) for 1 h and stimulation with 5 μg *S. cerevisiae *DNA/ml medium for 6 h (HKC) and 15 h (HaCaT cells). The results were normalised to 18S ribosomal RNA (ratio: target gene/housekeeping gene) and compared to non-treated samples (vehicle control) (* = p < 0.05; ** = p < 0.005 to positive control (SC-DNA transfected, non-inhibited); # = p < 0.05; ## = p < 0.005 to vehicle control).

To get a deeper indight into signal transduction mechanisms after adenoviral challenge, different signal transduction molecules (namely NFκB, ERK2, MAPKK, p38 MAPK, JAK/STAT, JNK and PI3K were inhibited and the IFN expression was compared. Results shown in Figure 3 were normalised to the vehicle control. The IFN-α/β regulation in non-inhibited positive controls was comparable to previous experiments (Figure 1). The HaCaT cells and HKC demonstrated involvement of the same signal transduction pathways for IFN-α/β production (Figure 3), except for the expression of IFN-α in HKC, which did not show any significant regulation of IFN-α in this experiment. Figure 3 indicates the involvement of p38 MAPK, PI3K, JNK and NFκB in triggering IFN-α/β production as the mRNA expression ratio was significantly decreased to a maximum difference of 27% compared to non-inhibited positive controls. Samples treated with Erk2/MAPKK and JAK/STAT inhibitors showed significantly reduced IFN-α/β expression from 46 to 56% compared to the positive control (Figure 3).

IFN-α mRNA levels of p38 MAPK-inhibited samples were significantly different compared to the non-inhibited positive control (p = 0.002) in HaCaT cells. The lowest signals in IFN-β expression were obtained in PI3K inhibited samples (13% in HaCaT cells (p = 0.05) and 18% in HKC (p = 0.01) compared to positive control and JNK blocked HKC (14%, p = 0.002).

### Investigation of adenovirus induced immune reaction *in vivo*

*In vitro *cell culture assays are not convenient for an investigation of systemic inflammatory effects after adenoviral challenge. To get some information about the systemic influences on vector reapplication, *in vivo *studies have been performed. In these experiments, immunocompetent (SKH-1^h/r^) and T-cell deficient athymic (Foxn-1^nu^) mice were twice transduced with the Ad-GFP vector. The transgene expression and inflammatory response was measured via life imaging and qRT-PCR.

**Figure 4:**
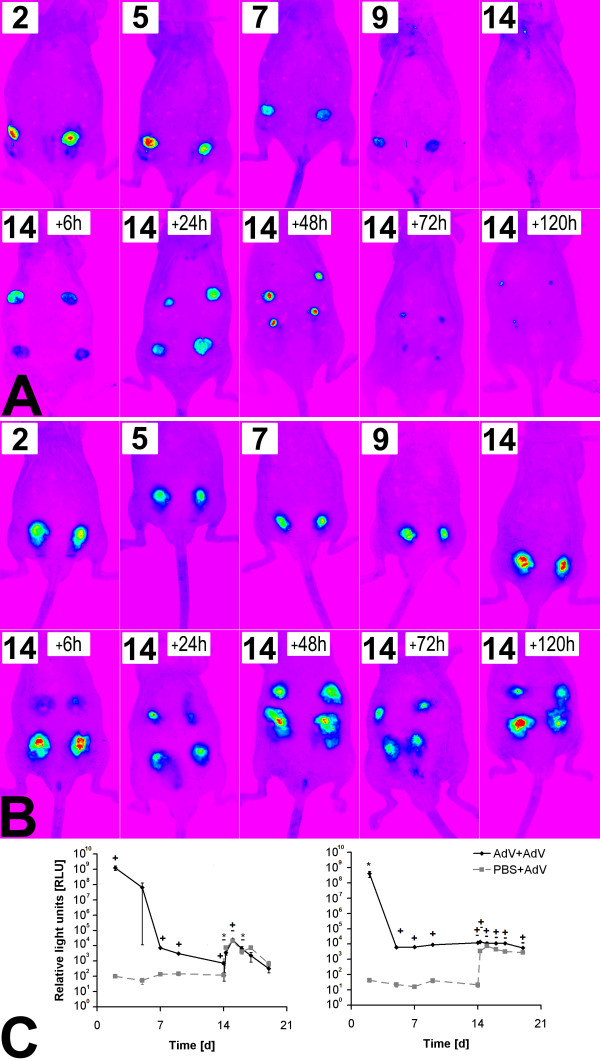
**Investigation of adenovirus induced transgene expression *in vivo***. Qualitative GFP fluorescence (relative light units; RLU) detection of immunocompetent (SKH-1^h/r ^(A)) and athymic (Foxn-1^nu ^(B)), hairless mice at timepoints 2 - 19 days after intradermal injection of 10^10 ^IU Ad-GFP at day 0 and reinjection of the same areas and transduction of a third area on day 14 (* = p < 0.05, + = p < 0.005 (AdV+AdV); - = p < 0.005 (PBS+AdV)). (C) Quantitative (RLU) GFP-fluorescence detection of an athymic (Foxn-1^nu^) and immunocompetent mice (SKH-1^h/r^) at timepoints 2 - 19 days after intradermal injection of 10^10 ^IU Ad-GFP at day 0 and reinjection of the same areas and transduction of two new areas on day 14.

An application of 10^10 ^IU Ad-GFP into immunocompetent mice resulted in a GFP expression detectable for 9 days with a maximum at day 2 (1,25 × 10^9 ^relative light units (RLU); Figure 4). A second injection of the same vector dose (AdV+AdV) as well as first transduction of two new areas (PBS+AdV) on day 14 led to an increased GFP expression for 5 days with a maximum of 2,4 × 10^4 ^RLU (AdV+AdV; p = 0.007) and 2 × 10^10 ^RLU (PBS+AdV; p = 0.007) after 24 h. An application of 10^10 ^IU AdGFP into athymic mice resulted in a GFP expression of 4 × 10^8 ^RLU two days post transduction. The expression level was reduced to 6 × 10^3 ^RLU five days post transduction and stayed on a relatively constant level until the end of the experiment on day 19 (p < 0.005). A second injection of the same vector dose into the same area did not show any changes in GFP expression. A transduction of the PBS treated (day 0) areas on day 14 resulted in an induction of GFP synthesis converging a level of 7.5 × 10^4 ^RLU after 24 h. No significant changes were measured at the following time points until the end of the experiment at day 19.

**Figure 5:**
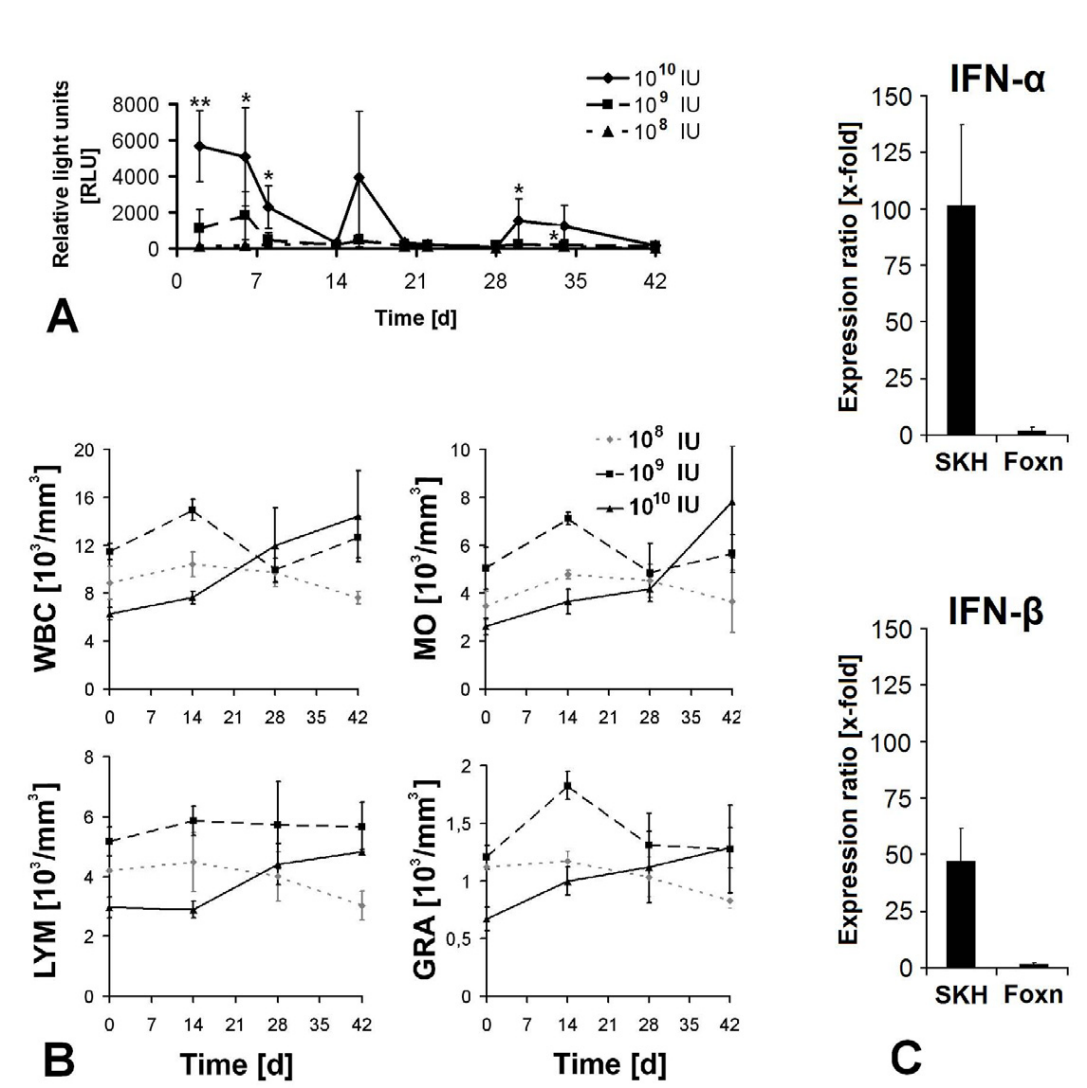
**Investigation of adenovirus induced immune reaction *in vivo***. (A) Quantitative GFP fluorescence and (B) numbers of leucocytes (WBC), granulocytes (GRA), lymphocytes (LYM) and monocytes (MO) in immunocompetent, hairless mice (SKH-1^h/r^) at timepoints of 14, 28 and 42 days after intradermal injection of 10^8 ^- 10^10 ^IU Ad-GFP. (C) RT-PCR analysis of IFN-α and IFN-β expression 48 h after intradermal injection of 10^10 ^IU Ad-GFP in immunocompetent (SKH) and athymic (Foxn) mice. Results were normalised to an untreated control area.

In addition to transduction with 10^10 ^IU AdGFP, two other virus concentrations of 10^8 ^IU and 10^9 ^IU were also tested for intradermal transduction of SKH-1^h/r ^mice. The application of 10^8 ^IU Ad-GFP did not show any detectable effects whereas the application 10^9 ^IU AdGFP lead to a GFP expression over a period of 7 days with a maximum of 1820 RLU on day 6 (p = 0.082). A reapplication of the same vector dose did not show any effect (Figure 5A). In contrast, the application of 10^10 ^IU Ad-GFP resulted in a GFP expression for 12 days with maximum at day 2 (5663 RLU, p = 0.094; Figure 5A). Reapplication of the same doses of Ad-GFP on day 14 and 28 reduced GFP expression [69% (p = 0.158) and 27% (p = 0.236) of expression on day 2] and duration (5 days; Figure 4). Blood count analysis showed a constant increase in numbers of leukocytes, granulocytes, lymphocytes and monocytes after application of 10^10 ^IU Ad-GFP; there were no significant differences after application of lower doses (Figure 5B). RT-PCR analysis of type-I-interferon expression after transduction of athymic and immunocompetent mice exhibited a lower expression of IFN-α (101-fold, p = 0.02 (SKH-1^h/r^); 1.8-fold, p = 0.09 (Foxn-1^nu^)) and IFN-β (47-fold, p = 0.1 (SKH-1^h/r^); 1.6-fold, p = 0.08 (Foxn-1^nu^)) in athymic compared to immunocompetent mice (Figure 5C).

**Figure 6:**
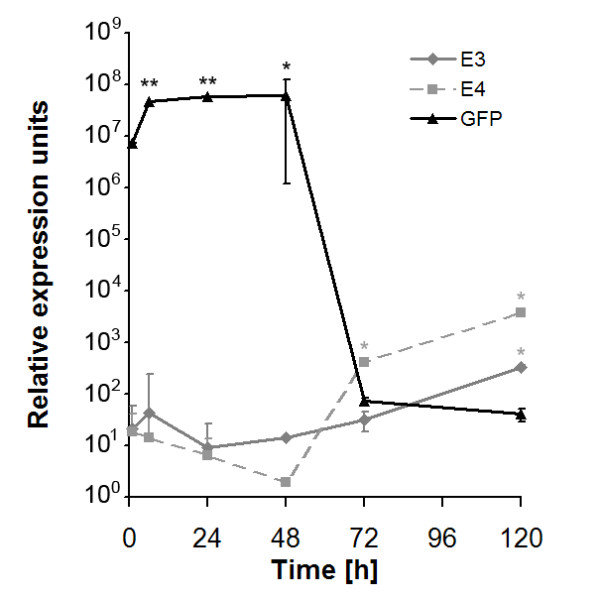
**Adenoviral mRNA-expression *in vivo***. RT-PCR analysis of viral regions E3, E4 and transgene GFP expression in immunocompetent mice up to 120 h post transduction with 10^10 ^IU Ad-GFP (* = p < 0.05, ** = p < 0.005). Data was normalised to 18S ribosomal RNA (ratio: target gene/housekeeping gene).

Additionally, the expression of other transcripts encoded by the adenoviral vector, was determined in this study. The viral early regions E3 and E4 are parts of the viral genome, possessing major regulatory functions. While, there was a strong decrease in GFP mRNA level in immunocompetent mice 48 h after second administration of the adenoviral vector, the viral early regions E3 and E4 showed an increasing expression for 120 h (Figure 6).

## Discussion

There are a number of studies that examine molecular mechanisms of innate immunity in mice, primates and humans[11,21-23,64]. However, these studies have a propensity to only address specialised antigen-presenting cells (APCs) such as DCs or MΦ[54,57] and RNA virus-induced immune reactions of APC[46,65,66]. In addition, data on the role of keratinocytes in innate immunity, particularly towards DNA internalization and DNA virus infection is limited. For optimization of a cutaneous gene delivery, it is critical to shed light on the molecular mechanisms a signalling pathways of the immunity of keratinocytes as they protect the body from the external environment. To address these issues, we have investigated the molecular mechanisms and signal transduction molecules involved in innate immunity towards DNA internalization into human keratinocytes. This study was performed using a replication incompetent, GFP-encoding adenovirus in order to significantly induce an innate immune response for an investigation of signal transduction molecules involved in adenovirus recognition.

This HaCaT cell line was used as it is simple to cultivate and experiments may be carried out under highly standardised and reproducible conditions[67]. Unlike other keratinocyte cell lines, the HaCaT cell acts like a primary keratinocyte under many experimental conditions[68]. The spontaneously immortalised human keratinocyte HaCaT cell line shows almost normal differentiation and keratinization in skin models[69] and was used throughout all experiments in comparison to primary keratinocytes.

To investigate differences between the HaCaT cell line and primary keratinocytes, this study examined the innate immune reaction after DNA stimulation. The results demonstrate the feasibility of both cell types for the investigation of innate immune responses. Cytokine expression in HaCaT displayed expected differences in comparison to HKC, but consistent with Köllisch *et al*., results were broadly comparable in chronology of interferon and cytokine induction at both cell types[70]. Any differences of induction intensity and kinetics between HaCaT cells and HKC were not unexpected due to altered growth conditions and the high number of passages of HaCaT cultures. Both cell types exhibit the same time-course with a primary induction of IFN-α (HaCaT: 12 h; HKC: 3 h), followed by an increased IFN-β expression (HaCaT: 15 h; HKC: 6 h) and cytokine expression (HaCaT: 24 h; HKC: 12 - 24 h).

Transduction of *ex vivo *full skin samples with an adenoviral vector also led to an induction of inflammatory mediators. This induction was measured on a comparable level to primary keratinocytes *in vitro*. This issue confirms the validity of data obtained in *in vitro *experiments with HKC.

Studies have described an activation of innate immunity independent of the species and sequence of internalised DNA, only the presence of nucleic acid in the cytoplasm elicits an immune reaction. The induction of type-I-interferon and cytokine synthesis during introduction of nucleic acids in the cytoplasm of APCs has been shown by different groups [48,53,55,56,71]. Limited data regarding immunogenicity of epidermal cells to different species of DNA is available. There is evidence for species independent induction of innate immunity when DNA is localised in the cytoplasm of a cell[48,49] and immune induction via cytoplasmatic localised mammalian DNA has been demonstrated especially by an organism's individual DNA[53,55,56,71].

This study demonstrates a high induction of type-I-IFN by (adenoviral) DNA internalization. The results suggest that cytoplasmic localised DNA triggers induction of type-I-interferon in HaCaT cells and HKC. In contrast to Shirota *et al*., we did not find any immune reaction after stimulation with DNA from calf thymus (CT) which does not support the hypothesis of immune induction through mammalian DNA[55]. For APC, Shirota *et al*. showed an 800-fold induction (DCs) and a 200-fold induction (MΦ) of IFN-β in consequence of CT-DNA internalization[55]. In contrast to mammalian DNA, fungal DNA stimulation led to a significantly increased expression of type-I-IFN as well as adenoviral DNA. Taken together, the results of the present study indicate an important role of keratinocytes in innate immunity. Keratinocytes may not recognise pathogens in the same manner as APCs, but the data proposes a major role of keratinocytes in innate immune defense against pathogens.

However, once induction of innate immunity by transfection of (adenoviral) DNA was demonstrated, this study investigated signal transduction pathways involved in DNA recognition. The Toll-like receptor family is the best known PRR with TLR-9 being the unique dsDNA detection receptor for this family[43]. Downstream signalling in TLR pathways is dependent on the adapter molecules MyD88 and TRIF[72]. Studies investigating DNA/RNA recognition in different cell types propose TLR-dependent and -independent pathways for DNA/RNA recognition in APCs[43,50,54,56,57]. There is limited data regarding molecular mechanisms of pathogen recognition in keratinocytes, so the involvement of TLR-9 and the adapter molecules for TLR signalling (MyD88 and TRIF) was investigated. The results indicate TLR-9 and TRIF-independent recognition of dsDNA in HaCaT cells and HKC. Interestingly, both cell types exhibit an upregulation of MyD88 after DNA internalization, which may denote involvement of other TLR receptors[73].

In addition to DNA recognition via TLR-9, there is also a possibility of RNA transcript or another pathogen associated molecular patterns (like zymosan, a polysaccharide from *S. cerevisiae*) induced TLR pathway induction. Therefore, this study investigated the induction of TLR-7 (viral RNA sensing receptor) and TLR-2 (zymosan detecting receptor). Evidence supports a RNA transcript and zymosan independent immune induction, since there were no changes in expression of these receptors detected. In contrast, and induction of DNA recognising receptor DAI was observed in both cell types, indicating a TLR-independent DNA recognition in keratinocytes.

Besides involvement of TLR receptor pathways in DNA recognition, this study examined participation of different effector molecules in cytokine induction. Inhibitor studies in HaCaT cell and HKC showed a 'two-step' inhibition in both cell types. On the one hand, an interferon down-regulation of 38 to 54% compared to a non-inhibited positive control was detected in consequence of blocking Erk2/MAPKK- and JAK/STAT-regulated pathways. On the other hand, inhibition of NFκB-, JNK-, p38 MAPK- and PI3K-regulated pathways led to a maximal IFN down-regulation of 25% compared to non-inhibited positive control. These results suggest a major participation of NFκB, JNK, p38 MAPK and PI3K and also an involvement of Erk2/MAPKK and JAK/STAT in signal transduction after recognition of cytoplasmatic localised DNA. PI3K-mediated signal transduction plays a major role in IFN induction in keratinocytes. In agreement with the present study, Philpott *et al*. described a PI3K-mediated TNF-α induction pathway[74]. A major involvement of JNK, p38 MAPK and NFκB in IFN-induction was not unexpected as these factors play a crucial role in initialization of transcription which is mediated by different pathways like TLR-, PI3K- JAK/STAT and MAPK-cascades[75]. Secondary involvement of Erk2/MAPKK- and JAK/STAT-regulated pathways may be due to a feedback mechanism mediated by cytokine receptors in the cells. The results suggest that induction of these pathways by cytokines may be primarily initiated by DNA recognition involving PI3K-dependent signalling cascades.

The data for dose-dependence between vector dose and immune reaction for *in vivo *adenoviral gene delivery is supported by other studies [54,55,57]. The results demonstrate that local and systemic immune reactions led to increased counts of leukocytes, lymphocytes, monocytes and granulocytes in mice.

The present study exhibits a comparable time course in GFP expression post reapplication of the identical vector dose into the same or non-transduced areas. These results suggest not only a local but also a systemic reaction against adenovirus, leading to decreased intensity and duration of transgene expression. Hence, therapeutic reapplication of the vector to stabilise transgene expression may be hindered[54,57]. Adenoviral *in vivo *transduction into athymic mice, which exhibit a deficient T-cell system and hence a reduced adaptive immune response, led to a stable transgene expression and reapplication of same vector doses into non-transduced areas showed an increase in transgene expression to the same level of areas of primary application. This, taken together with a diminished expression of type-I-interferon and proinflammatory cytokines, implies potential for effective adenoviral gene delivery into immunosuppressed skin and a future direction of our study may be to investigate inhibition of factors like type-I-interferons in order to optimise gene therapy with adenoviruses. Interestingly, there was a decrease in GFP mRNA level detected together with an increasing amount of viral region E3 and E4 mRNA (also encoded by the adenoviral vector), suggesting a gene silencing mechanism to regulate transgene expression in the tissue. Since this mechanism could not be explained yet, subsequent experiments are needed to analyse mRNA regulating effects.

The results of this study suggest an induction of the innate immunity triggered by cytoplasma localised DNA which is mediated by PI3K-, p38 MAPK-, JNK-, NFκB-, JAK/STAT- and ERK1/2-dependent pathways. After vector reapplication *in vivo*, immnocompetent mice possessed a decrease in duration and intensity in transgene expression. A stable transgene expression and a reduced inflammatory response in immunodeficient mice have been observed, suggesting opportunities for a higher efficiency of cutaneous gene delivery in immunosuppressed skin. It might be an interesting approach to influence in signal transduction cascades for an optimization of cutaneous gene delivery. Therefore, it is important to constitute any pathogen recognition receptors involved in recognition of adenoviral vectors. Since the present study did not observe any induction of TLRs 2, 7 and 9, but an induction of DAI, there is evidence for a TLR independent DNA recognition pathway in human keratinocytes. Further investigation will be required to fully describe and understand the complex mechanism in signal transduction after adenoviral challenge in human epidermal cells.

## Conclusion

In summary, the results of this study suggest an TLR-independent induction of the innate immunity triggered by cytoplasm localised DNA which is mediated by PI3K-, p38 MAPK-, JNK-, NFκB-, JAK/STAT- and ERK1/2-dependent pathways in human keratinocytes. This activation of innate immunity leads to a decrease in intensity and duration of adenovirally induced transgene expression. In contrast, a stable transgene expression and a reduced inflammatory response have been observed in immunodeficient mice. These results provide potential for an effective adenoviral gene delivery into immunosupressed skin.

## Methods

### Keratinocyte cell culture

Fresh human skin was obtained after abdominoplasty surgery (informed consent was given by the patient) and washed in PBS (PAA Laboratories, Coelbe, Germany). The skin was placed in a sterile petri dish and the hypodermis was excised. The skin was disinfected with Lavasept^® ^(Braun AG, Melsungen, Germany) for 5 min and washed with PBS, the tissue was sliced into pieces of 1 cm^2^. Skin pieces were transferred into a new petri dish with the epidermal side up and the skin was completely immersed with freshly prepared 0.2% dispase-solution (4.7 U/ml, Gibco, Paisley, United Kingdom [UK]) and incubated overnight at 4°C. The epidermis was peeled off and placed in Trypsin/EDTA-solution (0.05%/0.02%, Gibco, Paisley, UK) and reduced to pieces as small as possible. The pieces were incubated at 37°C for 20 min in a gently shaking (180 rpm) waterbath (GFL Burgwedel, Germany). The cell suspension was vortexed and the trypsin digestion was stopped by adding fetal bovine serum (FBS, HyClone, Logan, USA). The suspension was filtered through a 100 μm cell strainer (Becton Dickinson Heidelberg, Germany) and centrifuged at 400 × *g*, 20°C for 5 min. The cells were resuspended in a 5 ml keratinocyte medium (containing 3:1 Dulbecco's Modified Eagle Medium (DMEM, Gibco, Paisley, UK), Ham's F12 (Gibco, Paisley, UK), 10% FBS (Hyclone, Logan, USA), 1% Penicillin/Streptomycin (ICN, Aurora, USA), 4 mM L-Glutamin (ICN, Aurora, USA), 24.3 μg/ml Adenine (Calbiochem, Darmstadt, Germany), 5 μg/ml Insulin (Sigma, St. Louis, USA), 0.8 μg/ml Hydrocortisone (Calbiochem, Darmstadt, Germany), 1.346 ng/ml Triiodothyronine (Sigma, St. Louis, USA), 10^-6 ^M Isoproterenol (Sigma, St. Louis, USA), 20 ng/ml hEGF (Sigma, St. Louis, USA) and counted by CASY^®^-1 (Schärfe-System, Reutlingen, Germany). Cells were seeded at a density of 75,000 cells/cm^2 ^into collagen type I (Becton Dickinson Falcon, Heidelberg, Germany) precoated culture flasks. All different cell types including HaCaT (kindly provided by Prof. Fusenig, University of Heidelberg) cell lines were cultured at 37°C in humidified atmosphere of 5% CO_2_. HaCaT cells were cultured in DMEM containing 10% FBS (Hyclone, Logan, USA) and 1% Penicillin/Streptomycin, medium was changed every second day.

### Human full skin culture

Fresh, sterile human skin explants were obtained from three adult healthy patients (age range: 19-43 years) undergoing abdominoplasty surgery. The study was approved by the local ethics committee, and all of the patients gave written informed consent.

Immediately post excision, the skin was additionally washed in antiseptic (Octenisept, Schuelke-Mayr, Norderstedt, Germany) three times for ten seconds and three times in phosphate buffered saline (PBS). Subcutaneous fat was excised and the tissue was sliced into equilateral triangular pieces with sides of 2.5 cm length. These pieces were transferred to the base of a stainless steel chamber[76], placing the epithelial site upward. The upper part of the chamber was bolt down until the tissue was fixed, and during fixation, the skin explants were stretched to prevent contraction. Samples were cultured at the air-liquid interphase by using a 6-well plate filled with 5 ml of culture medium; DMEM, containing 10% FBS, 1% penicillin/streptomycin, and amphotericin B (25 μg/mL, PAA, Pasching, Austria). The tissue was incubated at 37°C in a humidified atmosphere containing 5% CO_2_, and the medium was changed twice a week.

Skin explants were prepared and cultured for at least 1 week. 10^10 ^IU Ad-GFP (green fluorescent protein) in 50 μl PBS were intradermally injected. After 12 h (n_PBS _= 3, n_Ad-GFP _= 3 samples per group; 3 patients per group), tissue biopsy specimens were harvested for total RNA isolation.

### Production and purification of recombinant adenovirus

In these studies, replication-deficient human *Δ*E1 adenoviruses type 5 (Ad5) with inserted cytomegalovirus (CMV)-promoter driven green fluorescent protein (GFP) was used. This virus exhibits a replacement of viral early E1 gene, which is essential for viral replication, with DNA material encoding for the green fluorescent protein (GFP). The virus was propagated in HER911 cells (DMEM containing 10% FBS and 1% P/S), purified by two sequential cesium chloride (CsCl_2_) gradients, and dialysed against 20 mM Tris/HCl, pH 8.0. The titer was determined using an Adeno-X™ rapid titer kit (Becton Dickinson Bioscience, K1653-1, Heidelberg, Germany). Virus stocks (2.11*10^11 ^infection units (IU)/ml) were stored at -80°C. Therefore, 10% glycerol was admixed.

### DNA purification

Adenoviral (Ad5) and bacterial (plasmid; P) DNA was purified using QIAamp DNA Mini Kit (Qiagen, Hilden, Germany) following the manufacturer's instructions for blood and body fluid. For purification of DNA from *Saccharomyces cerevisiae *(SC-DNA) (Deutsche Hefewerke, Nürnberg, Germany), 2 mg of cells were ground in a liquid nitrogen filled mortar. After cell disruption, 200 μl buffer ATL and 25 μl Proteinase K (Qiagen, Hilden, Germany) were added. The next steps were performed according to the manufacturer's instruction. The purified DNA was eluted in a final volume of 200 μl DNase-free H_2_O and the concentration was photometrically determined (Eppendorf Biophotometer, Hamburg, Germany) and stored at -80°C until usage. DNA samples were tested for contaminations via optical density (OD) ratio of 260 nm and 280 nm; DNA samples used for transfection showeded an OD ratio of >1.8. Calf thymus DNA was purchased from Sigma (Sigma, Taufkirchen, Germany) and bacterial plasmid DNA was purchased from Clontech (BD Biosciences Clontech, Heidelberg, Germany).

### Transfection

Cells were grown in 6-well plates until 90-100% confluency. DNA transfection complexes were prepared according to the manufacturer's instructions (Roche Molecular Biochemicals, Mannheim, Germany). Briefly, DNA was mixed 2:5 with the Fugene^® ^HD transfection reagent (Roche Molecular Biochemicals, Mannheim, Germany) in PCR-grade water (Roche Molecular Biochemicals, Mannheim, Germany) for 15 min at room temperature and then added to cells. Negative controls (vehicle controls) were treated with Fugene^® ^HD alone without DNA. Using this agent, a transfection efficiency of 22.4% (HaCaT cells) or 17.4% (HKC) has been reached using adenoviral DNA. If not mentioned otherwise, transfection experiments were performed in triplicate for each group.

### RNA isolation and analysis

Cultured cells were lysed directly with buffer RLT (Qiagen, Hilden, Germany) containing 1% (v/v) ß-Mercaptoethanol (Sigma, Taufkirchen, Germany). Isolation of total RNA was done using the RNeasy Mini Kit (Qiagen, Hilden, Germany), following the manufacturer's instructions for animal cells including DNA-digestion (RNase-free DNase Set, Qiagen, Hilden, Germany). Skin tissue has been taken and weighed in order not to exceed 30 mg. Tissue was stored at -80°C in RNAlater until further processing. Isolation of total RNA was done using the RNeasy Mini Kit, following the manufacturer's instructions for isolation of total RNA from heart, muscle and skin including DNA-digestion (RNase-free DNase Set, Qiagen, Hilden, Germany). RNA was eluted in a final volume of 30 μl RNase-free H_2_O. The concentration of RNA was determined using the Eppendorf Biophotometer (Eppendorf, Hamburg, Germany).

### Reverse Transcription

For transcription of RNA into DNA (reverse transcription) used for gene expression analysis via polymerase chain reaction (PCR), 1 μg of total RNA was transcribed into cDNA using the SuperScript™ II First Strand Synthesis System for RT-PCR (Invitrogen, Karlsruhe, Germany), following the manufacturer's instructions for first-strand synthesis using random hexamer primers. cDNA was stored at -20°C.

### Real-time PCR

Relative Quantification of mRNA was performed in a two-step real-time RT-PCR procedure using the fluorescent dye SYBR Green I (Light Cycler FastStart DNA Master SYBR Green I, Roche, Mannheim, Germany) and a Light Cycler 1.0 (Roche, Mannheim, Germany). The first step consisted of a RT reaction as described above, the second step of PCR amplification with specific primers (bottom). These primer pairs were validated to generate a single PCR-product. The PCR reactions were performed with 2 μl of cDNA, 0.5 μM of sense and antisense primers, 3 mM MgCl_2 _and 2 μl of FastStart SYBR Green reaction mix in a total volume of 20 μl. The cycling conditions were as follows: 95°C for 10 min at a ramp speed of 20°C/sec, 40 cycles (if not described otherwise) consisting of 94°C for 15 sec at a ramp speed of 20°C/sec, a primer specific annealing temperature for 10 sec at a ramp speed of 20°C/sec, 72°C for 10 sec at a ramp speed of 20°C/sec, followed by a melting point analysis: 95°C for 0 sec at a ramp speed of 20°C/sec, 65°C for 15 sec at a ramp speed of 20°C/sec, 95°C for 0 sec at a ramp speed of 0.1°C/sec, and finally a cooling phase: 40°C for 30 sec at a ramp speed of 20°C/sec. mRNA concentrations were corrected for 18S rRNA in each sample. Primer sequences for above mentioned genes are as follows: 18S sense 5'-gaaactgcgaatggctcattaaa-3'; 18S antisense 5'-cacagttatccaagtaggagagg-3' (annealing temperature (AT): 60°C); IFN-α sense 5'-acccacagcctggataacag-3'; IFN-α antisense 5'-ctctcctcctgcatcacaca-3' (AT: 60°C); IFN-β sense 5'-actgcctcaaggacaggatg-3'; IFN-β antisense 5'-agccaggaggttctcaacaa-3' (AT: 60°C); TLR-9 sense 5'-ggacctctggtactgcttcca-3'; TLR-9 antisense 5'-aagctcgttgtacacccagtct-3' (AT: 55°C); MyD88 sense 5'-gagcgtttcgatgccttcat-3'; MyD88 antisense 5'-cggatcatctcctgcacaaa-3' (AT: 55°C); TRIF sense 5'-ccagatgcaacctccactgg-3'; TRIF antisense 5'-ctgttccgatgatgattcc-3' (AT: 55°C). E3 sense 5'-cctgaaacacctggtccact-3' (AT: 56°C); E3 antisense 5'-ctgggtaaactcccgaatca-3' (AT: 56°C); E4 sense 5'-tcaggttgattcatcggtca-3' (AT: 56°C); E4 antisense 5'-cctaggcaggagggtttttc-3' (AT: 56°C); GFP sense 5'-acgtaaacggccacaagttc-3' (AT: 60°C); GFP antisense 5'-aagtcgtgctgcttcatgtg-3' (AT: 60°C), DAI sense 5'-aaagcatggacgatttaccg-3' (AT: 60°C); DAI antisense 5'- atgatgttcccgtgtccaat-3' (AT: 60°C).

### Protein inhibition

Prior to transfection, cells were pretreated with specific inhibitors (10 μM each) for NFκB (IκB-α inhibitor BAY117082 in DMSO), ERK1/2 (PD98059 in DMSO), p38 MAPK (SB203580 in DMSO), JNK (JNK II-inhibitor SP600125 in DMSO), PI3K (LY294002 in DMSO) and JAK-STAT (AG490 in EtOH) (Sigma, Steinheim, Germany).

### Animal studies

The research protocol described below conformed to all regulations related to animal use and other German federal statutes. It was performed in compliance with the 'Guide for the Care and Use of Laboratory Animals associated with the German Animal Welfare Act. Athymic mice (Foxn-1^nu^), which are deficient in T-cell system and provide a reduced adaptive immune reaction, were obtained from Harlan Winkelmann (Borchen, Germany) and immunocompetent, hairless "control" mice (SKH-1^h/r^) were obtained from Charles River (Sulzfeld, Germany) and housed under standard conditions. In the first experiment, a total of nine immunocompetent mice were randomised into three groups with n = 3 mice. Each animal was intradermally transduced with 10^8 ^- 10^10 ^infectious units (IU) Ad-GFP (green fluorescent protein) in 50 μl PBS at two discrete areas on the back. The transgene expression was controlled by the CMV promoter for high transgene expression rates. Transgene expression was localised and quantified every second day via Kodak Imaging Station 4000 MM (Kodak GmbH, Stuttgart, Germany). 14 and 28 days after first injection a second and third virus application was performed into the same areas and into a non-treated area. Blood samples were taken from the tail vein prior to virus application (see table 1).

**Table 1:** Inhibitors used in this study.

Bay11-7085	nuclear factor κB (NFkB)
**PD 98,059**	extracellular regulated kinase 2 (Erk2); mitogen-activated protein kinase kinase (MAPKK)

**SB203580**	p38 mitogen-activated protein kinase (p38 MAPK)

**LY294,002**	phosphoinositid-3-kinase (PI3K)

**Tyrphostin AG 490**	januskinase/signal transductor and aktivator of transcription (JAK/STAT)

**SP600125**	c-Jun N-terminal kinase (JNK)

In the second experiment, eighteen immunocompetent and eighteen immunodeficient mice (SKH-1^h/r ^and Foxn-1^nu^) were divided into six groups with n = 3 mice per group. Four distinct areas measuring 1 cm^2 ^were marked on the back of each mouse. On day 0, 10^10 ^IU Ad-GFP in 50 μl PBS or PBS alone was intradermally injected into two areas per mouse, followed by a second injection of 10^10 ^IU Ad-GFP into all four areas on day 14. 1, 6, 24, 48, 72 and 120 h after second injection, one group was euthanised by intrathoracic injection of 0.5 ml T61, the transduced skin areas were excised and stored in liquid nitrogen for RNA isolation.

### Statistical analysis

Differences were analysed for statistical significance with the Student's *t*-test. Error bars represent standard errors of the mean (SEM). RT-PCR analysis was displayed as expression ratio of treated and untreated (vehicle control) cells (x-fold expression).

## Abbreviations

**Ad**: adenoviral; **AdV**: adenovirus; **APC**: antigen presenting cells; **AT**: annealing temperature, **Ct**: calf thymus; **DC**: dendritic cells; **GFP**: green fluorescent protein; **HKC**: human primary keratinocytes; **IFN**: interferon; IL: interleukin; **IU**: infection units; **P**: plasmid; **PBS**: phosphate buffered saline; **PRR**: pathogen recognizing receptor; **RLU**: relative light unit; **SC**: Saccharomyces cerevisiae; **TLR**: toll-like receptor;

## Conflict of interest statement

The authors declare that they have no competing interests.

## Authors' contributions

MSc, LS and FJ conceived of and designed the study. MSc, MSo, SA-B, JS, JMO and MRK analyzed and interpreted the data. MSc and ADN drafted the paper and TH and HUS critically revised it for important intellectual content. All authors gave final approval of the version to be published.
